# Supplementary material for: 3′IsomiR Species Composition Affects Reliable Quantification of miRNA/isomiR Variants by Poly(A) RT-qPCR: Impact on Small RNA-Seq Profiling Validation
Source: Int J Mol Sci. 2023 Oct 21;24(20):15436. doi: 10.3390/ijms242015436 (PMC10607168; doi:10.3390/ijms242015436)
Supplement: Supplementary file 1 [file ijms-24-15436-s001.zip › ijms-2651167-supplementary.pdf]

Supplementary Figure S1

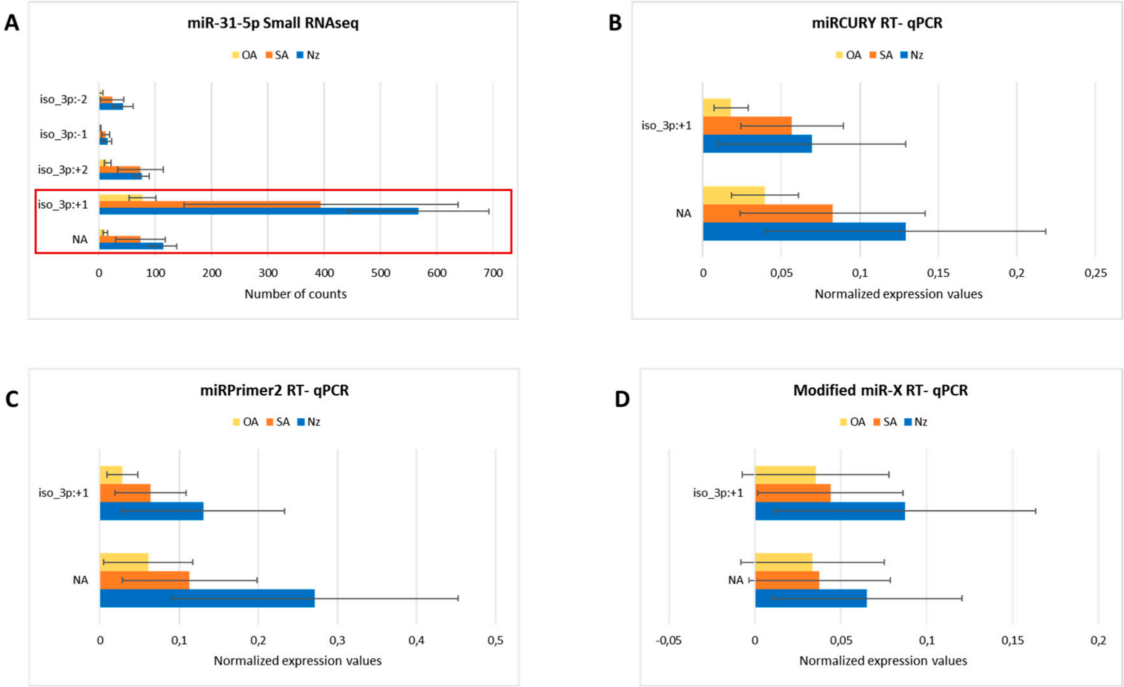

Expression values of miR-31-5p isoforms obtained when used small RNA-seq strategy (A) compared with the three RT-qPCR strategies (B,C,D) in seminal sEV biological samples

NA: miR-31-5p canonical sequence; iso_3p:+1: miR-31-5p 3' isomiR variant (3' addition G)

The red square in A highlights the two sequences of isomiRs (hsa-miR-31-5p and hsa-miR-31-5p iso_3p:+1) studied here.

Supplementary Figure S2

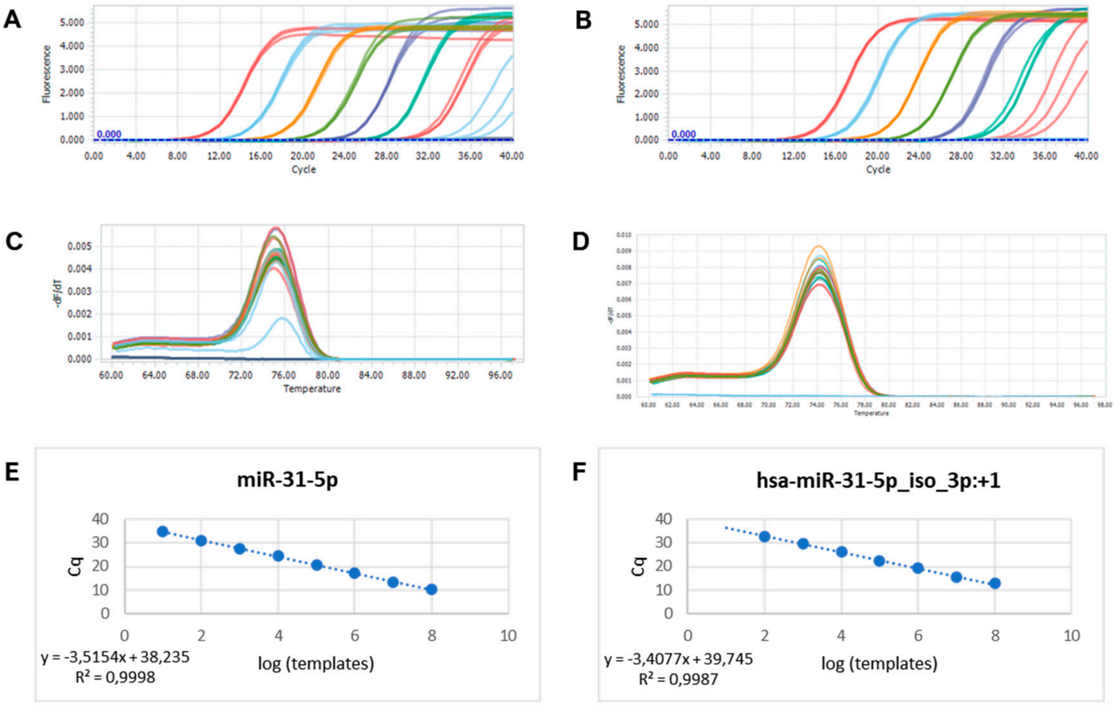

Synthetic cDNA assayed by qPCR miRPrimer2 strategy		
hsa-miR-31-5p assay		
isomiR ratio	Expected Cq values	Cq values
**100**|0	25,2	24,54
**75**|25	25,7	24,82
**50**|50	26,34	25,34
**25**|75	27,42	25,67
**0**|100	-	26,37

H

Synthetic cDNA assayed by qPCR miRPrimer2 strategy		
hsa-miR-31-5p_iso_3p:+1 assay		
isomiR ratio	Expected Cq values	Cq values
0|**100**	26,85	24,7
25|**75**	27,47	24,93
50|**50**	27,74	25,38
75|**25**	29,07	25,73
100|**0**	-	26,18

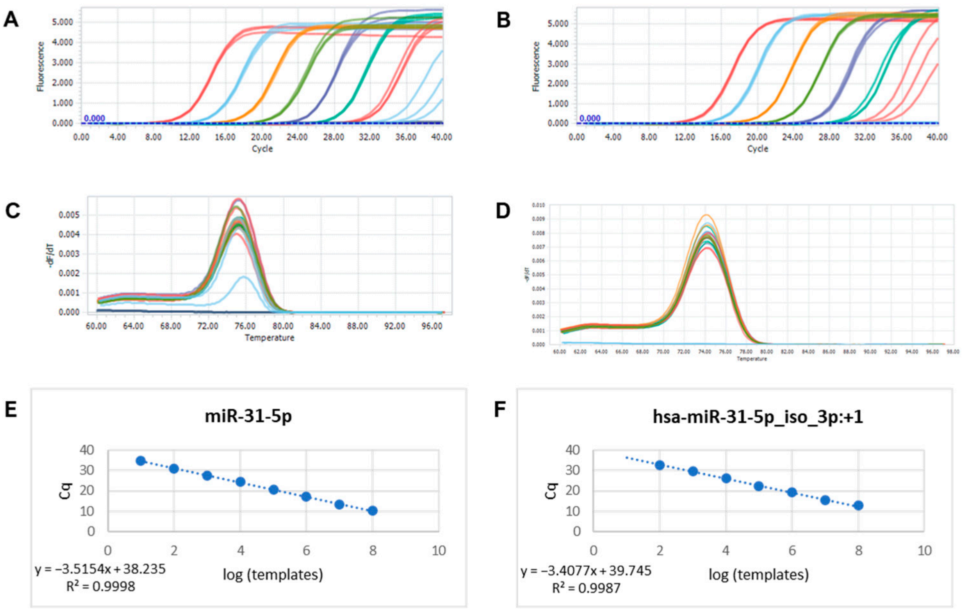

G

Synthetic cDNA assayed by qPCR miRPrimer2 strategy		
hsa-miR-31-5p assay		
isomiR ratio	Expected Cq values	Cq values
**100**|0	25.2	24.54
**75**|25	25.7	24.82
**50**|50	26.34	25.34
**25**|75	27.42	25.67
**0**|100	-	26.37

H

Synthetic cDNA assayed by qPCR miRPrimer2 strategy		
hsa-miR-31-5p_iso_3p:+1 assay		
isomiR ratio	Expected Cq values	Cq values
0|**100**	26.85	24.7
25|**75**	27.47	24.93
50|**50**	27.74	25.38
75|**25**	29.07	25.73
100|**0**	-	26.18

Standard curves for hsa-miR-31-5p (A,C,E) and hsa-miR-31-5p iso_3p:+1 (B,D,F) synthetic cDNA assayed by miRPrimer2 qPCR strategy, in order to determine the sensitivity of this qPCR methods. A, B) Amplification curves of eight log_{10} dilutions cDNA; C ,D) Melting curve analysis and E, F) Standard curves showed a consistent increase of Cq value for each step in the serial dilution (R^2: 0.99). Pooled synthetic cDNA solutions were also analysed to determine the specificity of the qPCR strategy (G, H). Cq value for each of the pooled solutions are shown together with the expected Cq value for each pool.

The different coloured lines correspond to amplification curves for serial dilutions of synthetic cDNA in A-D.

Supplementary Figure S3

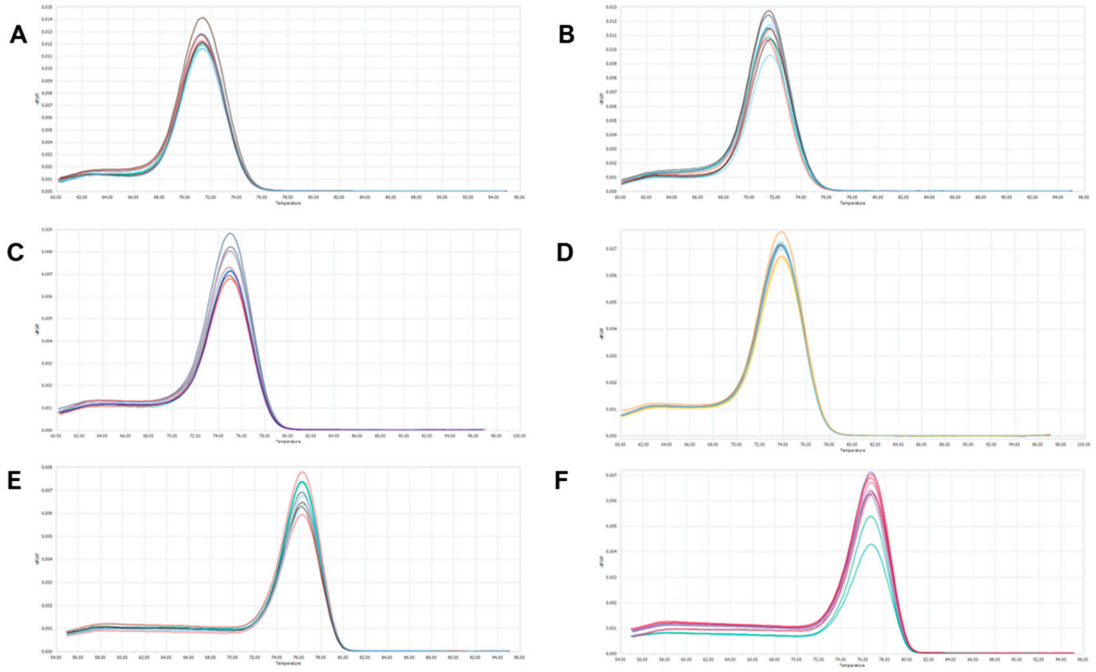

MiR-specificity determination in pools with different proportion of both hsa-miR-31-5p and hsa-miR-31-5p iso_3p:+1 synthetic RNA assayed by (A,B) miRCURY, (C,D) miRPrimer2 and (E,F) modified miR-X qPCR strategies. A, C, E) Melting curve analysis for hsa-miR-31-5p; B, D, F) Melting curve analysis for miR-31-5p iso_3p:+1.
